# Antibody-LGI 1 autoimmune encephalitis manifesting as rapidly progressive dementia and hyponatremia: a case report and literature review

**DOI:** 10.1186/s12883-019-1251-4

**Published:** 2019-02-07

**Authors:** Xuanting Li, Junliang Yuan, Lei Liu, Wenli Hu

**Affiliations:** 1 Department of Neurology, Beijing Chaoyang Hospital, Capital Medical University, Beijing, 100020 China; 2 Department of Neurology, Beijing Tongren Hospital, Capital Medical University, Beijing, 100730 China

**Keywords:** Autoimmune encephalitis, Limbic encephalitis, Leucine-rich glioma inactivated 1, Cognitive impairment, Hyponatremia, Arterial spin labeling

## Abstract

**Background:**

Anti leucine-rich glioma inactivated 1 (LGI1) encephalitis is a rare autoimmune encephalitis (AE), characterized by acute or subacute cognitive impairment, faciobrachial dystonic seizures, psychiatric disturbances and hyponatremia. Antibody-LGI 1 autoimmune encephalitis (anti-LGI1 AE) has increasingly been recognized as a primary autoimmune disorder with favorable prognosis and response to treatment.

**Case presentation:**

Herein, we reported a male patient presenting as rapidly progressive dementia and hyponatremia. He had antibodies targeting LGI1 both in the cerebrospinal fluid and serum, which demonstrated the diagnosis of typical anti-LGI1 AE. The scores of Mini-Mental State Examination and Montreal Cognitive Assessment were 19/30 and 15/30, respectively. Cranial magnetic resonance images indicated hyperintensities in bilateral hippocampus. The findings of brain arterial spin labeling and Fluorine-18-fluorodeoxyglucose positron emission tomography showed no abnormal perfusion/metabolism. After the combined treatment of intravenous immunoglobulin and glucocorticoid, the patient’s clinical symptoms improved obviously.

**Conclusions:**

This case raises the awareness that a rapid progressive dementia with predominant memory deficits could be induced by immunoreactions against LGI1. The better recognition will be great importance for the early diagnosis, essential treatment, even a better prognosis.

## Background

Autoimmune encephalitis (AE) is an infrequently and newly described group of neurological inflammation diseases related to specific autoantibodies. Various subgroups of AE are distinguished by these autoantibodies, which may lead to specific clinical presentations and different prognoses [1]. Among them, anti-leucine rich glioma inactivated 1 (LGI1) encephalitis is a treatable etiology of AE. Anti-LGI1 AE is characterized by cognitive impairment or rapid progressive dementia, psychiatric disorders, faciobrachial dystonic seizures (FBDS) and refractory hyponatremia [2, 3]. It is also considered a subtype of limbic encephalitis usually occurring without any detectable paraneoplastic cause [4, 5]. It is sensitive to the treatment of immunotherapy including steroids, intravenous immunoglobulin (IVIG) and other immunosuppression agents [6]. Unfortunately, it has often been misdiagnosed to be viral encephalitis or mental illness, which may delay immunotherapy and resulted in the deterioration of their conditions, including status epileptics and even coma [7].

Different from invasive fluorine-18-fluorodeoxyglucose positron emission tomography (^18^F-FDG PET), arterial spin labeling (ASL), without the use of intravenous gadolinium contrast, is highly sensitive technique to detect the changes of regional cerebral blood flow (CBF) [8, 9]. It was reported that a novel case of anti-N-methyl-d-aspartate receptor encephalitis was characterized by cerebral regional hyperperfusion on ASL [10]. To the best of our knowledge, there was only one case using the technique of ASL to detection and follow-up of perfusion changes in anti-LGI1 AE [11].

Herein, we reported a 56-year-old man presenting as rapidly progressive dementia and hyponatremia with anti-LGI1 AE, and described the clinical manifestations, imaging findings of ASL, and treatment and outcomes. As far as we know, this is the second report using the combination of ^18^F-FDG PET and ASL to explore the metabolism changes in anti-LGI1 AE.

## Case presentation

A 56-year-old man presented with fever for three weeks and memory decline for two weeks, especially deficits in anterograde amnesia. Initial neurological examination revealed rapidly progressive cognitive impairment. The scores of Mini-Mental State Examination (MMSE) and Montreal Cognitive Assessment (MoCA) were 19/30 and 15/30, respectively. No epileptic seizures occurred during the disease course.

**Fig. 1:**
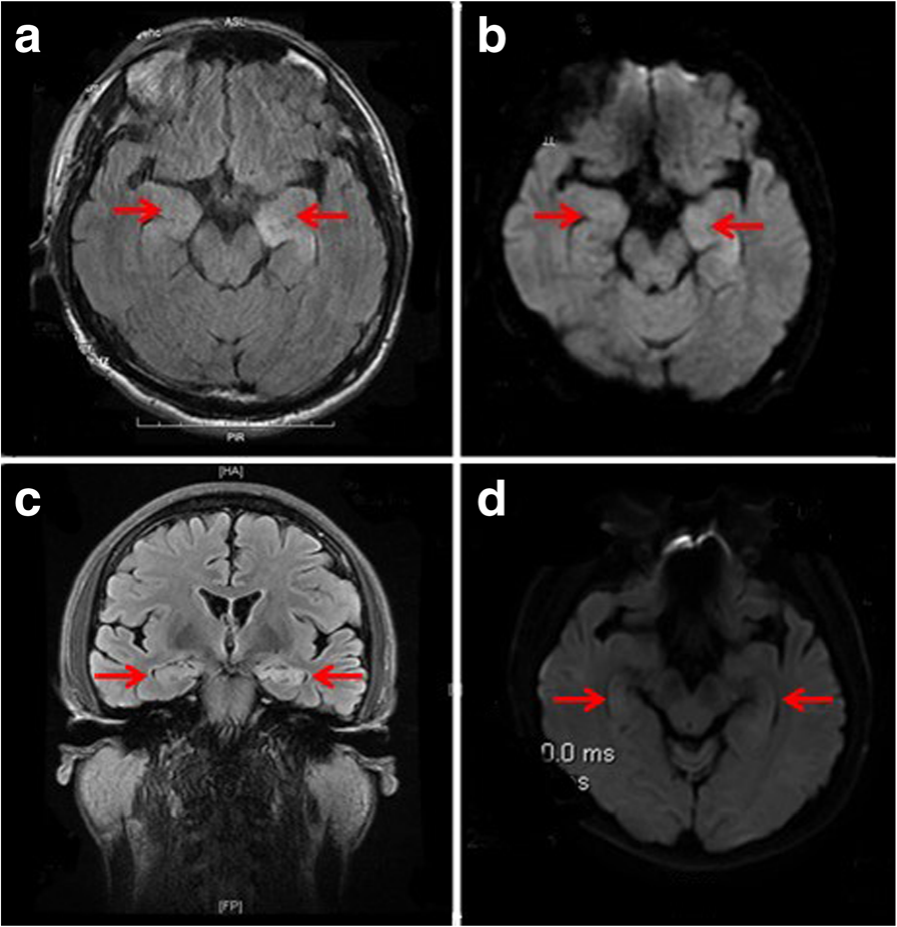
Cranial magnetic resonance images (MRI) of this patient. T2-weighted fluid-attenuated inversion recovery (**a**) and the corresponding plane in diffusion weighted imaging (**b**) sequences showed hyperintensities of bilateral hippocampus. Repeated MRI showed some abnormal hyperintensities particularly in the left hippocampus 12 days after the initial MRI scan (**c**, **d**)

**Fig. 2:**
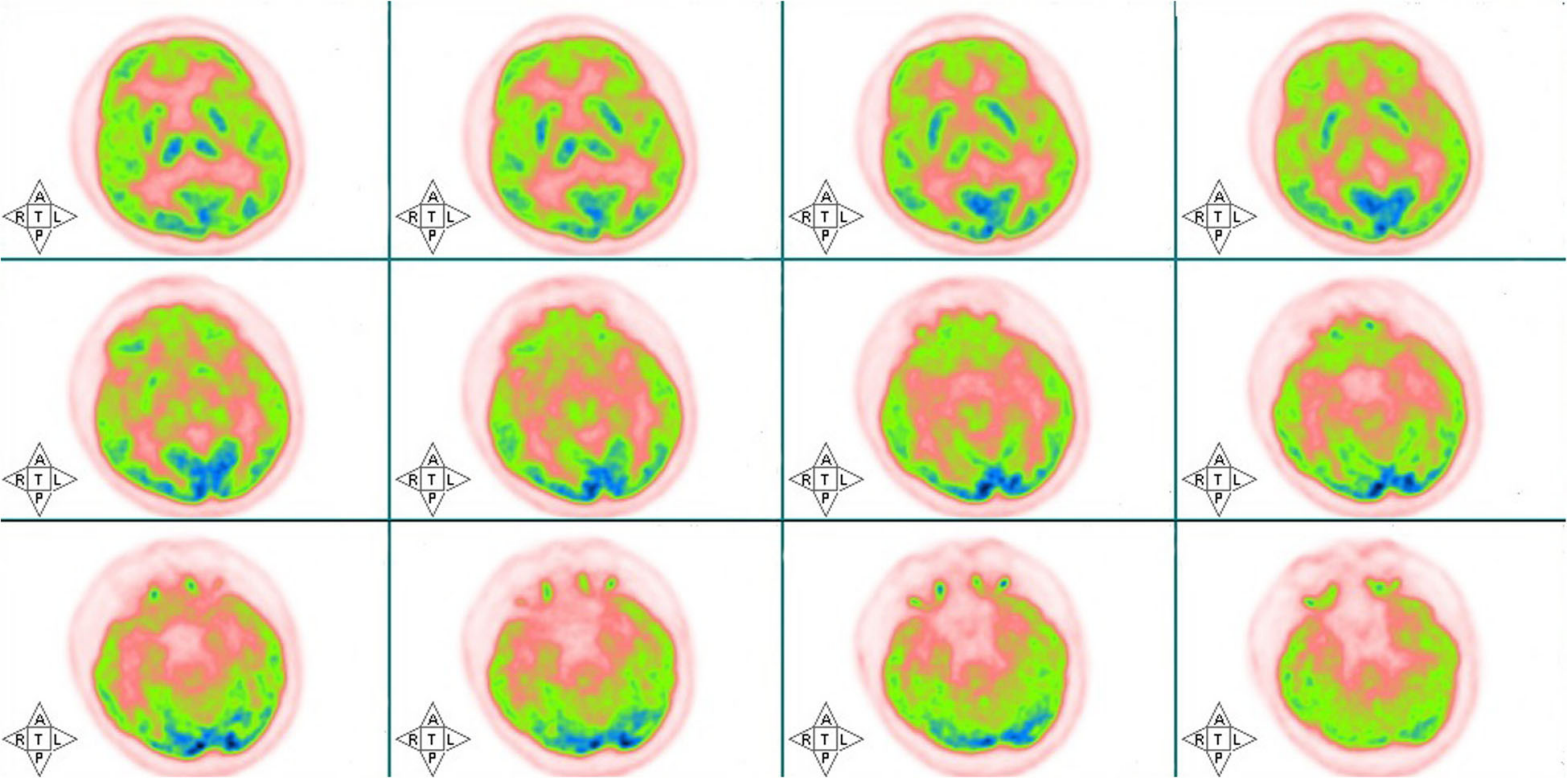
Fluorine-18-fluorodeoxyglucose positron emission tomography showed no abnormal metabolism in the brain

**Fig. 3:**
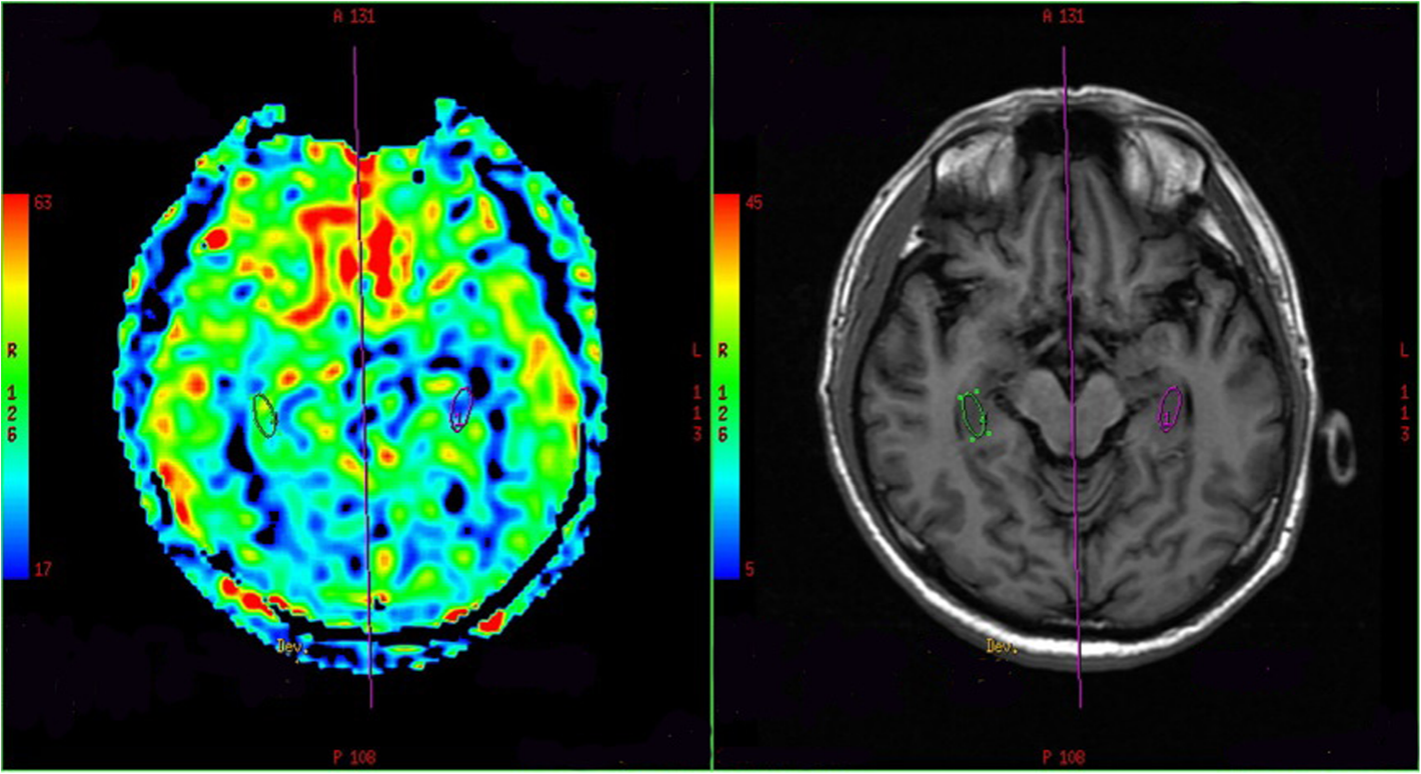
Arterial spin labeling showed no abnormal perfusion in bilateral hippocampus

The cerebrospinal fluid (CSF) showed mildly elevated leukocyte (19/uL, normal range 0–8/uL) and glucose (5.39 mmol/L, normal range 2.5–4.5 mmol/L), lowered chloride (113.5 mmol/L, normal range 120-130 mmol/L), and a normal protein level (44 mg/dL, normal range 20–40 mg/dL). At the same time, the serum tests of sodium, chloride and blood glucose were 126.1 mmol/L, 94.2 mmol/L and 7.26 mmol/L, respectively. The LGI1-Ab was positive (+++) both in the serum and CSF (Fig. 1), however, the other biomarkers of AE (NMDAR-Ab, AMPAR2-Ab, GABA_B_R-Ab, Caspr2-Ab), tumor markers (CEA, AFP, CA125, CA19–9, CA15–3, CA724, SCCAg, NSE, T-PSA, CYFRA21-1) and paraneoplastic neuronal antibodies (anti-Hu, −Ri, -Yo, −Ma/Ta, -Amphiphysin, -CV2, -SOX1, −Tr) were all unremarkable. The other laboratory tests revealed within normal limits. Electroencephalogram was normal. Cranial magnetic resonance images (MRI) indicated hyperintensities in bilateral hippocampus on T2-weighted fluid-attenuated inversion recovery (Fig.1a) and diffusion weighted imaging (Fig.1b) sequences. Twelve days later, the repeat MRI showed some abnormal hyperintensities particularly in the left hippocampus (Fig.1c, d). Chest computed tomography and ^18^F-FDG PET showed no signs of tumor (Fig.2). One month after onset of cognitive decline, the findings of ASL and ^18^F-FDG PET showed no abnormal perfusion/metabolism in the bilateral hippocampus (Fig.3).

He was diagnosed with anti-LGI1 AE, with the treatment of methylprednisolone and IVIG, later with oral prednisone for six months. Fifteen days after his admission, he recovered obviously and discharged from our department with mild memory impairment. During 30 days’ follow-up, his symptoms were in complete remission with immunomodulation. The cognitive function became normal with MMSE 30/30.

## Discussion and conclusions

Herein, we described a case presenting as rapidly progressive dementia and hyponatremia. The clinical manifestations, positive LGI1-Ab both in CSF and serum, neuroimaging findings, the treatable effect and favorable prognosis contributed to the diagnosis of anti-LGI1 AE. Thorough differential diagnosis of AE should be considered in patients with presentation of symptoms, such as memory impairment.

In spite of rare condition, anti-LGI1 AE is manifesting with memory deterioration, FBDS, epileptic seizures, mental disorders and hyponatremia. The hyponatremia is a characteristic feature of anti-LGI1 AE, and 60%~ 88% of such patients have refractory hyponatremia according to the prior studies [3, 7, 12, 13]. The pathogenic mechanism is likely associated with the syndrome of inappropriate antidiuretic hormone secretion causing by the simultaneous LGI1 expression of the hypothalamus and kidney [14, 15]. Our case was consistent with prior reports, with the lower level of sodium 126.1 mmol/L, however, the hyponatremia in our case was refractory.

Besides hyponatremia, cognitive impairment is also one of common neurological disorders in anti-LGI1 AE. Memory disorder, especially short memory impairment, is the most prominent [16]. Up to 15% of the anti-LGI1 AE patients developed rapidly progressive cognitive dysfunction, which further increase the difficulty of the differential diagnosis with neurodegenerative disorders [17]. However, the neuroimaging features and long-term cognitive prognosis are still not well understood. The cognitive impairment of anti-LGI1 AE patients may be caused by the structural damage of hippocampal memory system [18]. Some Japanese scholars found that the influence of the interaction of LGI1-ADAM22-AMPAR on the long-term depression and long-term potentiation may play a significant part in the process of memory dysfunction [19]. Fortunately, this cognitive impairment might be prevented by early immunotherapy. The treatment with high-dose steroids, IVIG and/or plasma exchange was considered to be first-line therapy, however, the clinical remission upon methylprednisolone and IVIG does not prove therapeutic efficacy of these drugs [20].

^18^F-FDG PET is a useful tool to help evaluate the treatment response of encephalitis [21]. It is newly used to support the diagnosis of AE, characterized by the hypometabolism on the parietal and occipital cortices and the hypermetabolism on the basal ganglia [21]. Another PET study also found that markedly increased FDG uptake was initially revealed in both limbic system during the acute phase of disease, and gradually decreased and eventually returned to normal after treatment [21]. Different from invasive ^18^F-FDG PET, ASL is a noninvasive tool with high sensitivity to changes in regional CBF [8, 9]. It’s proposed that the areas of hyperperfusion on ASL is in accordance with the reginal enhancement in the acute and subacute inflammation processes [22]. To the best of our knowledge, there was only one case using the technique of ASL on detection and follow-up of perfusion changes in anti-LGI1 AE [11]. This case described anti-LGI1 AE with hyperperfusion in hippocampus and amygdala during the acute stage, and follow-up neuroimaging proved improvement after the treatment [11].

As for our patient, there are some strengths should be addressed. Firstly, the clinical features, the neuroimaging findings, the positive LGI1 antibodies both in CSF and serum, and the hyponatremia, were all contributed to the diagnosis of anti-LGI1 AE. Secondly, our case presented with obvious memory impairment, after the immunomodulation, the cognitive disorders fully recovered. We inferred abnormal structures in bilateral hippocampus might be associated with memory disorder. Thirdly, the refractory hyponatremia was a distinguishing feature in our case, highlighting the higher prevalence of hyponatremia in patients with anti-LGI1 AE. Fourthly, ASL is a highly sensitive tool to detect the changes of regional CBF, especially in acute stage. However, ASL and ^18^F-FDG PET of our case showed no abnormal perfusion/metabolism in the brain. This may be due to the fact that ASL and PET were performed four weeks after the onset and not in the acute phase. As far as we know, this is the second report using the combination of ^18^F-FDG PET and ASL to explore the metabolism changes in anti-LGI1 AE. Further prospective studies with larger sample sizes will be needed to utilize ASL to validate such findings.

In summary, AE is rare and the causes are still largely unknown. It is an under-recognized condition and favorable prognosis. Our case increases the awareness of anti-LGI1 AE, as early recognition and treatment is critical and necessary. Our case also highlights the importance of differential diagnosis and ongoing follow-up of patients with rapidly progressive dementia. Randomized clinical trials are needed in anti-LGI1 AE.

## Abbreviations

^18^F-FDG PET: Fluorine-18-fluorodeoxyglucose positron emission tomography
AE: Autoimmune encephalitis
ASL: Arterial spin labeling
CBF: Cerebral blood flow
CSF: Cerebrospinal fluid test
FBDS: Faciobrachial dystonic seizure
IVIG: Intravenous immunoglobulin
LGI1: Leucine-rich glioma inactivated 1
MMSE: Mini-Mental State Examination
MoCA: Montreal Cognitive Assessment
MRI: Magnetic resonance image
